# Development of reaching during mid-childhood from a Developmental Systems perspective

**DOI:** 10.1371/journal.pone.0193463

**Published:** 2018-02-23

**Authors:** Laura Golenia, Marina M. Schoemaker, Egbert Otten, Leonora J. Mouton, Raoul M. Bongers

**Affiliations:** University of Groningen, University Medical Center Groningen, Center for Human Movement Sciences, Groningen, The Netherlands; University of Exeter, UNITED KINGDOM

## Abstract

Inspired by the Developmental Systems perspective, we studied the development of reaching during mid-childhood (5–10 years of age) not just at the performance level (i.e., endpoint movements), as commonly done in earlier studies, but also at the joint angle level. Because the endpoint position (i.e., the tip of the index finger) at the reaching target can be achieved with multiple joint angle combinations, we partitioned variability in joint angles over trials into variability that does not (goal-equivalent variability, GEV) and that does (non-goal-equivalent variability, NGEV) influence the endpoint position, using the Uncontrolled Manifold method. Quantifying this structure in joint angle variability allowed us to examine whether and how spatial variability of the endpoint at the reaching target is related to variability in joint angles and how this changes over development. 6-, 8- and 10-year-old children and young adults performed reaching movements to a target with the index finger. Polynomial trend analysis revealed a linear and a quadratic decreasing trend for the variable error. Linear decreasing and cubic trends were found for joint angle standard deviations at movement end. GEV and NGEV decreased gradually with age, but interestingly, the decrease of GEV was steeper than the decrease of NGEV, showing that the different parts of the joint angle variability changed differently over age. We interpreted these changes in the structure of variability as indicating changes over age in exploration for synergies (a family of task solutions), a concept that links the performance level with the joint angle level. Our results suggest changes in the search for synergies during mid-childhood development.

## Introduction

The development of goal-directed reaching is important as reaching is involved in many manual everyday life actions. Refinement of reaching skills occurs during mid-childhood development (5- to 10-years of age), a relevant developmental period which has often been overlooked. Until now, the few studies that did examine mid-childhood development have solely focused on the performance level of reaching; showing for example that reaching movements become more accurate and less variable with increasing age [1–4] and that reaching movements can be better adjusted to sudden changes in target location [5–9]. Doing so, these studies did not emphasize the contribution and development of other levels of analysis involved in reaching, such as for instance the joint level (see for an exception Schneiberg et al. [10]). This might be attributed to the theoretical approach underlying these studies, such as the information processing approach or the computational neuroscience perspective (i.e., internal models and representations). Following these approaches, studies tacitly assumed that there is a single process (i.e., feedback/feedforward mechanisms) or component (i.e., representation) in the system that controls motor behavior [2,11–13]. This idea implicitly entails that developmental changes in reaching follow directly from developmental changes in the single process or component. That is why examining changes in performance over age was considered sufficient to understand developmental changes. Even though these studies and approaches have gathered important knowledge about mid-childhood development, we propose that if one wants to understand the full range and complexity of developmental changes, one should depart from studying just one level and should distinguish the development of individual levels contributing to reaching (see for detail criticism of earlier studies Golenia et al. [14]).

A perspective that views changes in motor behavior not as a reflection of changes in a single process or component, but as resulting from the interaction of multiple changing components acting on different levels of the system is the Developmental Systems (DS) perspective [15–18]. Importantly, the system is not confined to the body, but includes the full action-perception cycle. Automatically, this means that the environment and the task are equally important parts of the system. The DST´s starting point is that over development each component of the system changes on its own time-scale. This means that the current behavioral state of the system results from the interaction among all components. Hence, if one or multiple components change, the behavior might change. From this perspective a great deal of understanding about the acquisition of reaching skills during early-infancy has been achieved [19–23]. Here, we noted the lack of using ideas from the DS perspective on the study of reaching development during mid-childhood [14]. Following the DS perspective, understanding developmental trends requires examining not only variables at the performance level, but also variables at other levels and their relation. We took a first step in filling in this gap by focusing on development at the level of the joint angles in the arm and how it relates to the development at the performance level (i.e., kinematics of movements of the end-point, which is in the current study the tip of the index finger).

Joint angles are defined as the relative orientations of the different segments of the arm and hand (finger, wrist, elbow and shoulder joints). The wrist has for example two joint angles: the wrist can flex or extend and abduct or adduct. An important characteristic of the joint angles is that they are abundant [24]. This means that there are more joint angle combinations available than necessary to accomplish the task of reaching (i.e., reaching the 3D target position in space with the tip of the index finger) [24–26]. For example, imagine sitting in front of a table and keeping your index finger tip at one position on the table. It is possible to move your arm while keeping the index finger tip on the same position on the table, meaning that you use different joint angle configurations that all accomplish this task. Thus, abundance allows for variability in joint angles over repetitions of reaching trials. The main goal of this study is to examine how this variability in joint angles changes during mid-childhood when performing a reaching task and how it affects the performance of the endpoint at the reaching target, i.e. endpoint position variability. Studying such variability, and in particular the structure within it, is generally considered an important way to reveal underlying developmental processes [27–31].

How can structure in joint angle variability be assessed? An often used approach in the literature is to parse joint angle variability over repetitions of trials into two parts [24,26,32]: (1) One part is the joint angle variabilty that does not affect task success, meaning that joint angles co-vary to stabilize the endpoint around its mean position (goal-equivalent variability). This part of the joint angle variability is the variability demonstrated in the earlier example of moving the arm while keeping the index finger tip at one position on the table. (2) The other part is the joint angle variability that causes the endpoint to deviate from its mean position, shifting the end-point away from the mean position (non-goal-equivalent variability). This variablity over repetitions of trials therewith results in error around the mean position, usually seen in endpoint position variability around the target. Examining the structure in joint angle variability allows to examine the relation between joint angles and the index finger position in spatial measures at the reaching target. We used the Uncontrolled Manifold (UCM) method to quantify the structure in joint angle variabilty [24,26,32–34]. The UCM method partitions variability in joint angles over repetitions of trials into goal-equivalent variability (GEV) and non-goal-equivalent variability (NGEV). Higher levels of GEV than NGEV correspond to a relatively invariant, stable value of the performance (i.e., index finger position).

An additional goal of this study was to evaluate whether and how the availability of visual information about the arm influenced the structure in joint angle variability. The DS perspective states that the behavior of the system results from the interaction of not only the components of the person, but also from the environment and task [35]. An important environmental component involved in reaching movements is visual information about the hand and arm. Previous developmental studies that focused on the performance of the reach found that vision influenced end-point error (i.e., more errors in no vision conditions) and even influenced the developmental trend [1,2,13]. It could also impact joint angle variability because of the required reliance on proprioceptive information when no visual information is available. We therefore asked whether vision availability is an equally large constraint in reaching at each age during development.

In line with the DS perspective, we focused on changes in the relation of the performance level and the joint angle level in goal-directed reaching movements during mid-childhood development. We do so because even though index-finger movements are brought about by joint angles, this does not mean that there is a direct relation between the two; only the structure in the variability can characterize such relation. We therefore examined the developmental trend of the structure in joint angle variability across 6-, 8-, and 10-year-old children and adults. By that we aimed, in line with the DS perspective, to increase the understanding of reaching by using a level-overarching explanation. Doing so, we focused on developmental trends of spatial variables of endpoint position variability (i.e. constant and variable error), joint angle variability (i.e. standard deviation) and structure in joint angle variability (GEV and NGEV) at the end-point of the movement. From earlier studies, we know that both the constant and variable error decrease with age [13,36]. NGEV should relate to the error variables of the index finger because this is the joint angle variability that influences the index finger position. We therefore expected a decrease in NGEV over age. GEV, on the other hand, does not influence the index finger position, meaning that the developmental trend of this variable does not have to be related to the trend of the performance of the index finger. We proposed two competing hypotheses for GEV: Either GEV decreases with age or GEV stays similar across age groups.

## Method

### Participants

40 typically developing children, recruited from local sport clubs and schools around the university, and 15 young adults participated (age range in years/months = 19/2–28/5). Children were distributed in three groups based on their age resulting in a group of twelve 6-year-olds (age range in years/months = 5/9–6/5), a group of fifteen 8-year-olds (age range in years/months = 7/6–8/6) and a group of thirteen 10-year-olds (age range in years/months = 9/7–10/5). All children and adults were right-handed.

### Ethics statement

The local ethics committee of the Center for Human Movement Sciences (University Medical Center Groningen) approved the study that was conducted according to the principles expressed in the Declaration of Helsinki. Adult participants and children’s parents or legal guardians provided written informed consent prior to the experiment.

### Movement-ABC-2 test

The Movement Assessment Battery for children-2^nd^ Edition (MABC-2) was performed by all children prior to the experiment to test their motor development [37]. The MABC-2 test provides an indication of motor functioning across fine and gross motor tasks for children aged 3 to 16 years. The test consists of three age-related item-sets, measuring manual dexterity (three items), aiming and catching (two items), and balance (three items). Children get a score on each item, which are then transformed into standard scores, ranging from 1–19. A percentile equivalent for the total test score is used as outcome measure which ranges from 0.1 to 99.9. A typical development is indicated by a score above the 16^th^ percentile. Adults received no motor assessment, because the MABC-2 has no norm for adults. Instead adults were asked whether they had any neurological diseases, recent injuries or musculoskeletal problems in the neck, shoulder, arm or hand regions, which was not the case.

### Apparatus

**Fig 1:**
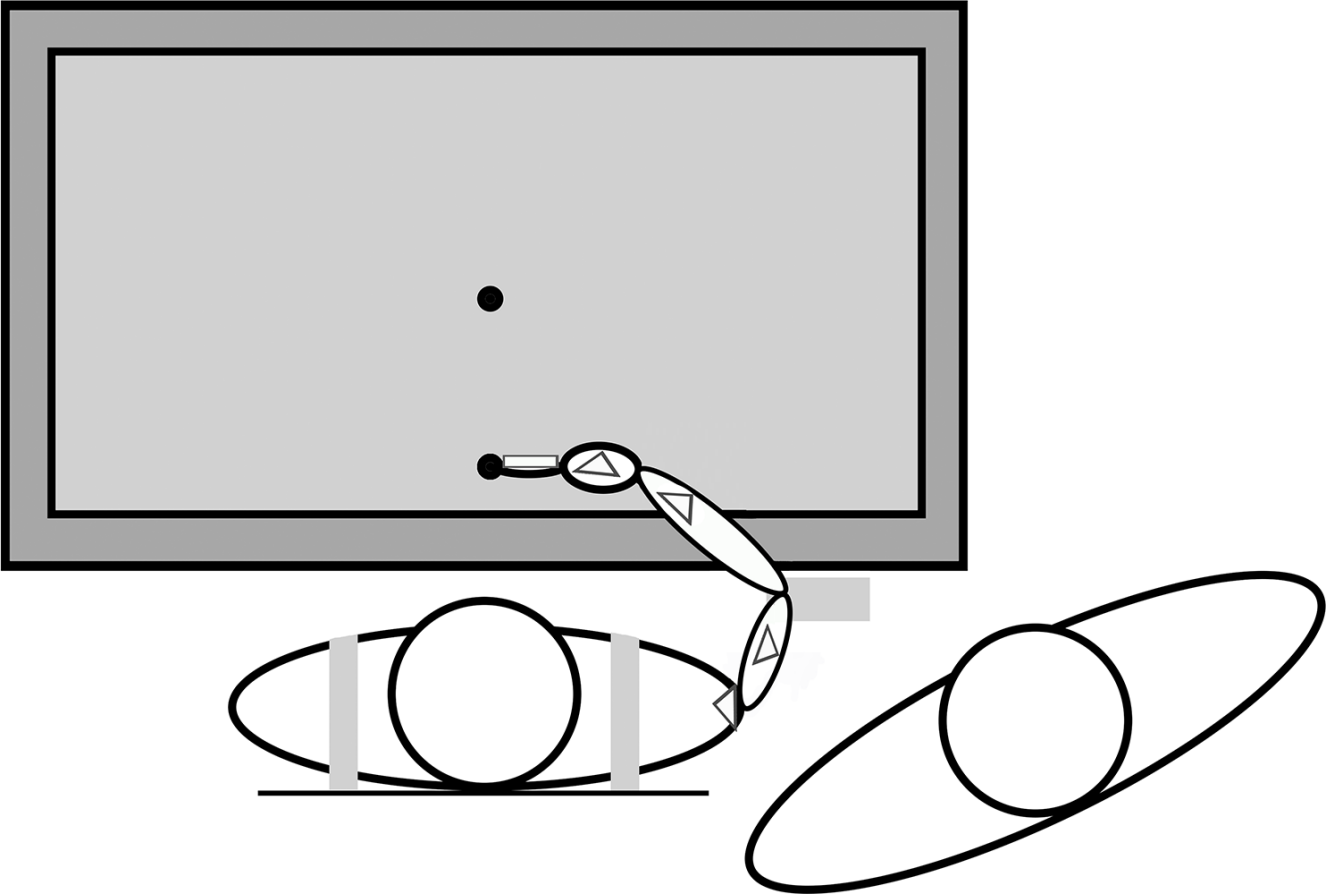
Experimental setup. Bird’s-eye view on a participant sitting at the experimental table. The participant was gently strapped to the chair (grey straps). The posture represents the start position of each trial. The elbow of the participant was placed on the elbow rest and the tip of the index finger was positioned on the start position. If the participant was a child, an experimenter was sitting next to the child. Triangles and the rectangle on the finger represent rigid bodies (the rigid body on the sternum cannot be seen).

3D position data of all segments of the right arm were collected with two Optotrak 3020 system sensors (Northern Digital, Waterloo, Canada). To obtain joint angles of the shoulder, elbow, wrist, and index finger, six rigid bodies (each with three LED markers) were placed on the participants’ right arm and the trunk [38]. Five triangular shaped rigid bodies were attached to the sternum, to the flat part of the acromion, to the upper arm just below the insertion of the deltoid, to the lower arm proximal to the ulnar and radial styloid, and to the dorsal surface of the hand (Fig 1). The sixth rigid body was placed on the index finger so that it splinted the finger to prevent motion of the inter-phalangeal joints (i.e., the finger was considered as one segment in the analysis). Two different sizes were used; matching the length of the index finger of adults and children, respectively. Nineteen bony landmarks were digitized using a standard pointer device [38] and were linked to the position of the LED markers on the rigid bodies.

Fig 1 shows the experimental setup. The task was performed at a black table (height = 72 cm), in which a large television screen (Panasonic, 62*111 cm) was horizontally mounted presenting the task display that was developed using Presentation (Neurobehavioral systems, Berkely, CA). Lighting of the room could be controlled to manipulate visual feedback of the arm and hand. Participants sat in a chair adjusted to their height and the length of their arm, so that the elbow had a 90-degree angle and was at the same height as the table, keeping the relation between table and participant similar across participants. For children, we used a chair (Tripp Trapp, Stokke, Sweden) of which also the plate for the feet could be adjusted so that children could sit with their legs resting on this plate. The back of the chair was extended in height with a board so that participants’ trunk could gently be strapped against it to prevent movements of the torso, but allowing free movements of the shoulder and elbow joints. To keep the start posture of the upper extremity as similar as possible over trials, the olecranon of the right arm had to be placed on a marked location on an elbow rest that was positioned at a comfortable height on the right side of the participant (Fig 1).

### Procedure and design

After anthropometrics were measured, markers were attached and participants were seated at the table. Prior to testing, participants completed three practice trials to be sure that participants understood the instructions. In the case of a child, an experimenter sat next to the child to ensure that the hand and the arm were in the required position at the beginning of each trial, and that the child was attentive to the task (Fig 1).

At the beginning of each trial the start location was illuminated (red dot, 1cm diameter) and participants were instructed to touch the start location with the tip of their right index finger while the elbow was positioned on the elbow rest (elbow left the rest to reach the target). A target location (green dot, 2cm diameter) appeared and participants pointed as quickly and accurately as possible to the target location, according to instructions. The trial ended with holding the tip steady on the target location for a short period of time. The start location (located 10 cm away from the body) and target location were displayed at the midline of the screen in the depth direction (which was aligned to the body midline). Reaching distance was 30% of the average arm-length of norm values of the concerned age group (18.5cm in 6-year-olds, 20.5cm in 8-year-olds, 22.5cm in 10-year-olds, and 28.0cm in adults, according to Gerver & De Bruin [39].

Reaching movements in two conditions were performed: (1) Reaching to the target with normal room lighting so that visual information about the position of the arm was available (vision condition) and (2) reaching to the target in the dark so that the position of the arm and hand could not be seen (no-vision condition). Note, the illuminated target was visible in both conditions.

In total, the experimental session consisted of 60 trials (30 in each condition). We needed this high number of trials to get a proper approximation of the uncontrolled manifold (see next section) under the assumption that children’s behavior was rather variable. Participants completed two blocks of 15 trails in each condition that were separated by short breaks (around 3 minutes). Blocks were presented in a random order.

### Data analysis

For all analysis, customized data analysis programs were developed in Matlab (MathWorks; Natick, Massachusetts). To determine the initiation and the termination of the reaching movement, a backward (movement initiation) and forward (movement termination) search was performed from the maximum in the velocity profile of the forward direction (x-direction) of the index finger until a threshold of 5 cm/s, respectively. The first points below threshold were taken as the initiation and termination of the reaching movement, respectively. Note that all dependent variables were analyzed at the instant of movement termination because reaching the target was the only constraint in the current study.

All variables were analyzed with repeated measures ANOVAs using SPSS version 20.0 (IBM, Armonk, New York). All repeated measures ANOVAs had age-group (6, 8, 10-year-old children, and adults) as between-subject factor. For the factor age-group we were interested in the developmental trend, therefore we tested linear, quadratic, and cubic contrasts. Note, the age intervals between age-groups were not similar, i.e., the interval between 10-year-old children and the adults was much larger than the age interval between the other age groups. Therefore, a linear statistical trend does not mean that the developmental trend over ages is also linear. We took this into account when interpreting the results. We also report the omnibus test of the factor age-group for completeness. If the assumption of sphericity was violated, the Greenhouse–Geisser correction was applied. The level of significance was set at *α* < 0.05. Generalized eta-squared, *η*^*2*^_*G*_, [40] was used to calculate effect sizes, and interpreted according to Cohen's recommendation of 0.02 for a small effect, 0.13 for a medium effect, and 0.26 for a large effect [41]. Only results with an effect size larger than 0.02 were reported. For the three children groups, a one-way ANOVA was conducted to test effects of age on the M-ABC percentile scores.

#### Performance level

For each trial, the constant error (CE; mean difference between target position and tip position of the index finger at movement termination) and the variable error (VE; within-subject standard deviation of CE) was calculated. Even though we focused primarily on spatial variables, we also calculated movement time (MT; time from movement initiation to movement termination). These variables were analyzed with a repeated measures ANOVA with visual condition (vision, no-vision) as within-subject factor and the polynomial contrasts of age-group. Moreover, to ensure that the results regarding spatial variability (main outcome measures) were not confound by differences over age in speed-accuracy tradeoff, we calculated linear regressions between MT and accuracy for each individual participant. The intercept and the slope of the regression lines were analyzed with a repeated measures ANOVA with visual condition (vision, no-vision) as within-subject factor and age-group as between subject factor.

#### Joint angle level

We examined the following joint angles of the right arm: shoulder plane of elevation (SPE), shoulder elevation (SE), shoulder inward–outward rotation (SIOR), elbow flexion–extension (EFE), elbow pronation–supination (EPS), wrist flexion–extension (WFE), wrist abduction–adduction (WAA), index finger flexion–extension (FFE), and index finger abduction–adduction (FAA). Joint angles were calculated as proposed in the ISB standardization proposal for the upper extremity by Wu et al. [42]. Standard deviation (SD) of the joint angles at movement termination over 30 repeated reaching trials was calculated. For each joint angle, a repeated measures ANOVA on joint angle SD was performed with visual condition (vision, no-vision) within-subject factor and the polynomial contrasts of age-group.

#### Structure in joint angle variability

The UCM analysis was performed as described previously [24,26,33,43]. The UCM analysis was computed for the moment of movement termination from 30 reaching trials (N). Elemental variables were defined as the joint angles of the shoulder, elbow, wrist, and finger resulting in a 9-degree of freedom (DoF) system. The position of the endpoint was selected as performance variable (3-DoF). The relation between changes in elemental variables and changes in the performance variable were computed based on a 3D forward kinematics model and united in a Jacobian (J) matrix [33,43]. Its null-space was used as a linear approximation of the UCM. The variance components GEV and NGEV were computed by projecting the total variance in joint space onto the null-space of J and the orthogonal complement, respectively. Eqs 1 and 2 show the computation of GEV and NGEV, where *tr* denotes the trace of a matrix, *J* denotes the Jacobian matrix, *C* denotes the covariance matrix of all joint angles, *n* denotes the dimension of the joint space (n = 9) and *d* denotes the dimension of the task space (d = 3). GEV and NGEV were normalized by its number of DoFs.

$$GEV=\frac{{tr(null(J)}^{t}*C*null(J))}{n-d}$$ Eq (1)

$$NGEV=\frac{{tr((({J^{t})}^{⊥})}^{t}*C*({J^{t})}^{⊥})}{d}$$ Eq (2)

To correct for non-normal data distribution, GEV and NGEV were log transformed prior to the statistical analysis [44] and are displayed before log transformation in the figures in the results section. A repeated measures ANOVA was performed with visual condition (vision, no-vision) and variability (GEV, NGEV) as within-subject factors and age-group as between subject-factor. To check that the findings at movement termination did not originate from differences at movement initiation, we compared GEV and NGEV at movement initiation and termination. We conducted two separate repeated measures ANOVA´s for GEV and NGEV with vision (vision, no-vision) and time (initiation, termination) as within-subject factor and age-group as between subject factor.

## Results

365 out of 2700 trials were removed from the dataset because markers were not recorded due to occlusion, so that at least one variable could not be determined. This left 2335 trials that were used for analysis. Group averages of anthropometrics as well as the M-ABC scores of the children groups are presented in Table 1. All children scored above the 16^th^ percentile of the M-ABC *(M* = 64.5%, range = 25.0–99.5%) indicating that all children were typically developing. M-ABC percentile scores were similar among age groups (*p* = 0.429).

**Table 1:** Anthropometric data of all age groups and M-ABC scores of children groups.

	6-year-olds	8-year-olds	10-year-olds	Adults
Mean age in yrs (range)	5.9 (5.7–6.5)	8.1 (7.6–8.6)	10.2 (9.7–10.5)	22.8 (19.2–28.5)
Gender (*n* male/ *n* female	6/6	9/6	7/6	7/8
Mean height (*SD*) in mm	120.7 (5.6)	133.1 (6.6)	145.0 (6.9)	183.3 (10.0)
Mean weight (*SD*) in kg	21.4 (2.3)	26.6 (3.2)	34,5 (5.9)	72.2 (11.8)
Mean arm length (*SD*) in cm	49.3 (3.2)	54.2 (2.8)	61.4 (3.4)	78.2 (6.1)
Mean M-ABC percentile (range)	57.5 (25–99)	69.5 (25–98)	65.2 (25–84)	-

### Performance level

**Fig 2:**
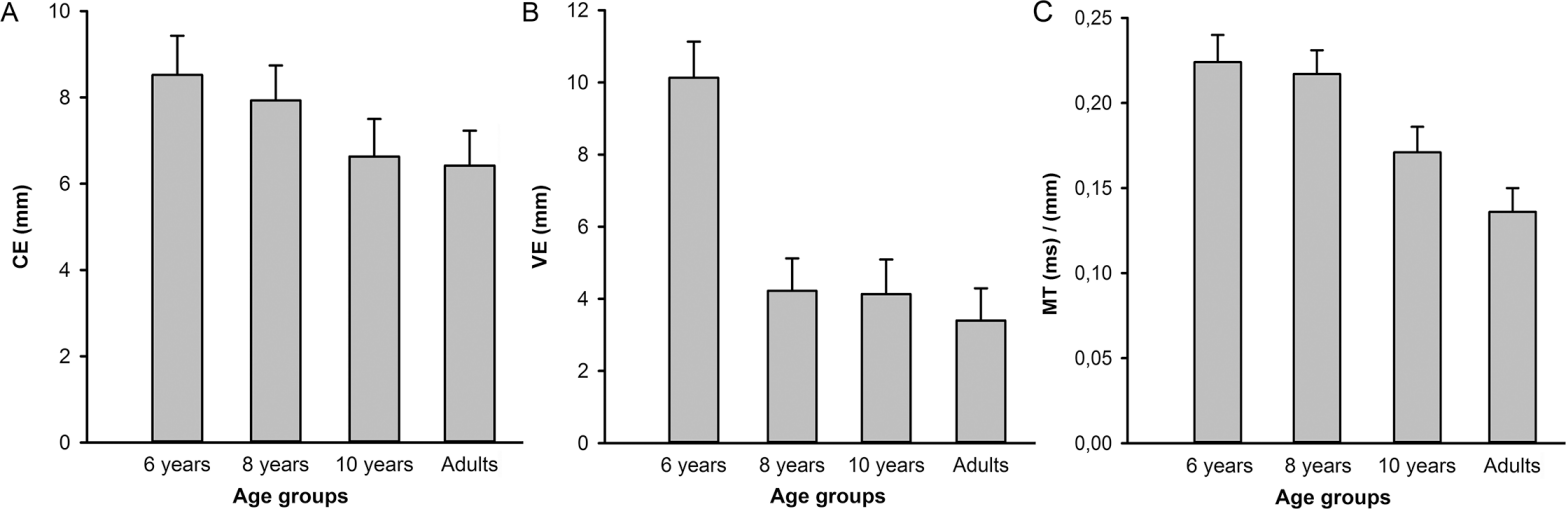
Error values at movement termination for each age group. A. Constant error (CE). B. Variable error (VE). C. Movement time (MT). The mean of the vision and no-vision condition is presented. Error bars represent SEM.

The age-group effect of CE was not significant, however as Fig 2A indicates CE decreased from 8.5mm in 6-year-old-children to 6.4mm in adults. The linear contrast for CE was negative and marginal significant (contrast estimate *=* -1.69, *p* = .054). VE decreased with age, which was revealed by a significant age-group effect, *F*(3,51) = 10.06, *p* < .001, *η*^*2*^_*G*_ = 0.21. Contrast analysis revealed a significant negative linear effect (contrast estimate *=* -4.51, *p* < .001), and a quadratic effect (contrast estimate *=* 2.57, *p* = .008). As can be seen in Fig 2B, both effects seem to emerge from the sharp decrease in VE from 6-year-olds to 8-year-olds. MT decreased over age (Fig 2C), indicated by a significant age-group effect, *F*(3,51) = 16.72, *p* < .001, *η*^*2*^_*G*_ = 0.48. A significant negative linear contrast was found for MT (contrast estimate *=* -0.069, *p* < .001). CE, VE and MT showed no vision effect.

Concerning the speed-accuracy tradeoff, the intercepts of the regression line between MT and accuracy revealed a significant age-group effect, *F*(3,51) = 8.78, *p* < .001, *η*^*2*^_*G*_ = 0.27, demonstrating that the intercept decreased over age (6-year-olds: *M* = 0.24, *SEM* = 0.02; 8-year-olds: *M* = 0.20, *SEM* = 0.01; 10-year-olds: *M* = 0.18, *SEM* = 0.02; adults: *M* = 0.14, *SEM* = 0.01). The slope of the regression line revealed no age-group effect (*p* = .27), showing no differences between groups in the tradeoff between speed and accuracy.

### Joint angle level

**Fig 3:**
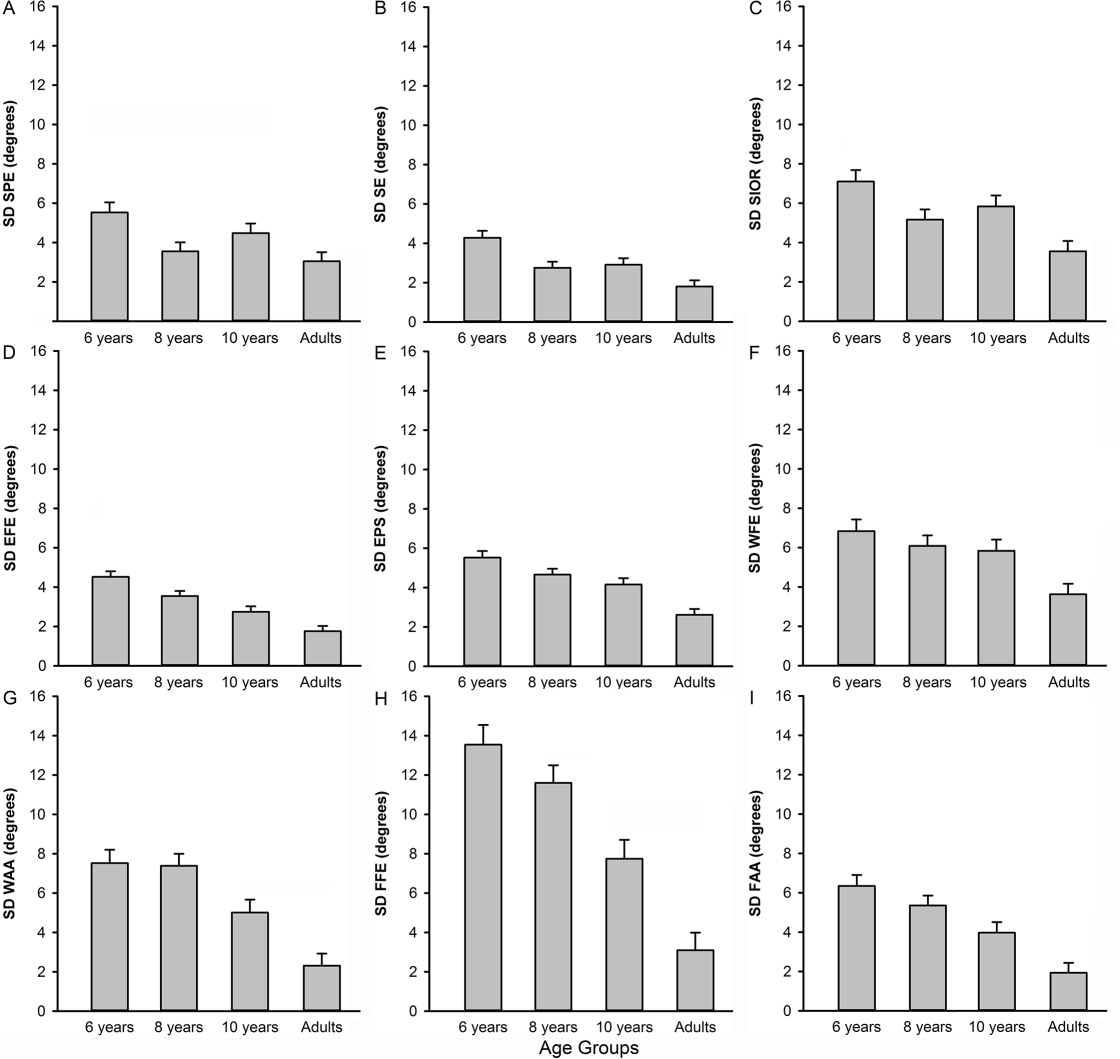
Joint angle standard deviation for the moment of movement termination per joint angle for each age group. A. SPE, B. SE, C. SIOR, D. EFE, E. EPS, F. WFE, G. WAA, H. FFE, I. FAA. The mean of the vision and no-vision condition is presented. Error bars represent SEM.

Fig 3 shows the joint angle SD at movement termination for all joint angles and age-groups. SD of all joint angles revealed a significant main effect of age-group (Table 2). All three shoulder joint angles (SPE, SE, SIOR) showed a significant negative linear effect (*p´s* = < .001–0.004) and a cubic effect (*p´s* = < .017–0.049) for age-group. As seen in Fig 3A, 3B and 3C, joint angle SD of the shoulder joint angles decreased from 6-year-olds to 8-year-olds, but increased in 10-year-olds (but SD values did not reach the those of the 6-year-olds), followed by a decrease to adult level. Polynomial contrasts of all elbow, wrist and finger joint angles (Fig 3D–3I) revealed negative linear effects (all *p´s* = < .001). As seen in Fig 3D–3I, the joint angle SD decreased from 6-year-olds to adults. No effects for vision were found for SD of any of the joint angles.

**Table 2:** Main effect of age for joint angle standard deviation per joint angle.

Joint angle	F	df	p-value	*η*^*2*^_*G*_
SPE	5.04	3,51	.004	0.20
SE	9.31	3,51	< .001	0.27
SIOR	7.28	3,51	< .001	0.26
EFE	18.17	3,51	< .001	0.46
EPS	15.36	3,51	< .001	0.39
WFE	6.35	3,51	< .001	0.21
WAA	15.30	3,51	< .001	0.43
FFE	24.62	3,51	< .001	0.55
FAA	13.35	3,51	< .001	0.39

### Structure in joint angle variability

**Fig 4:**
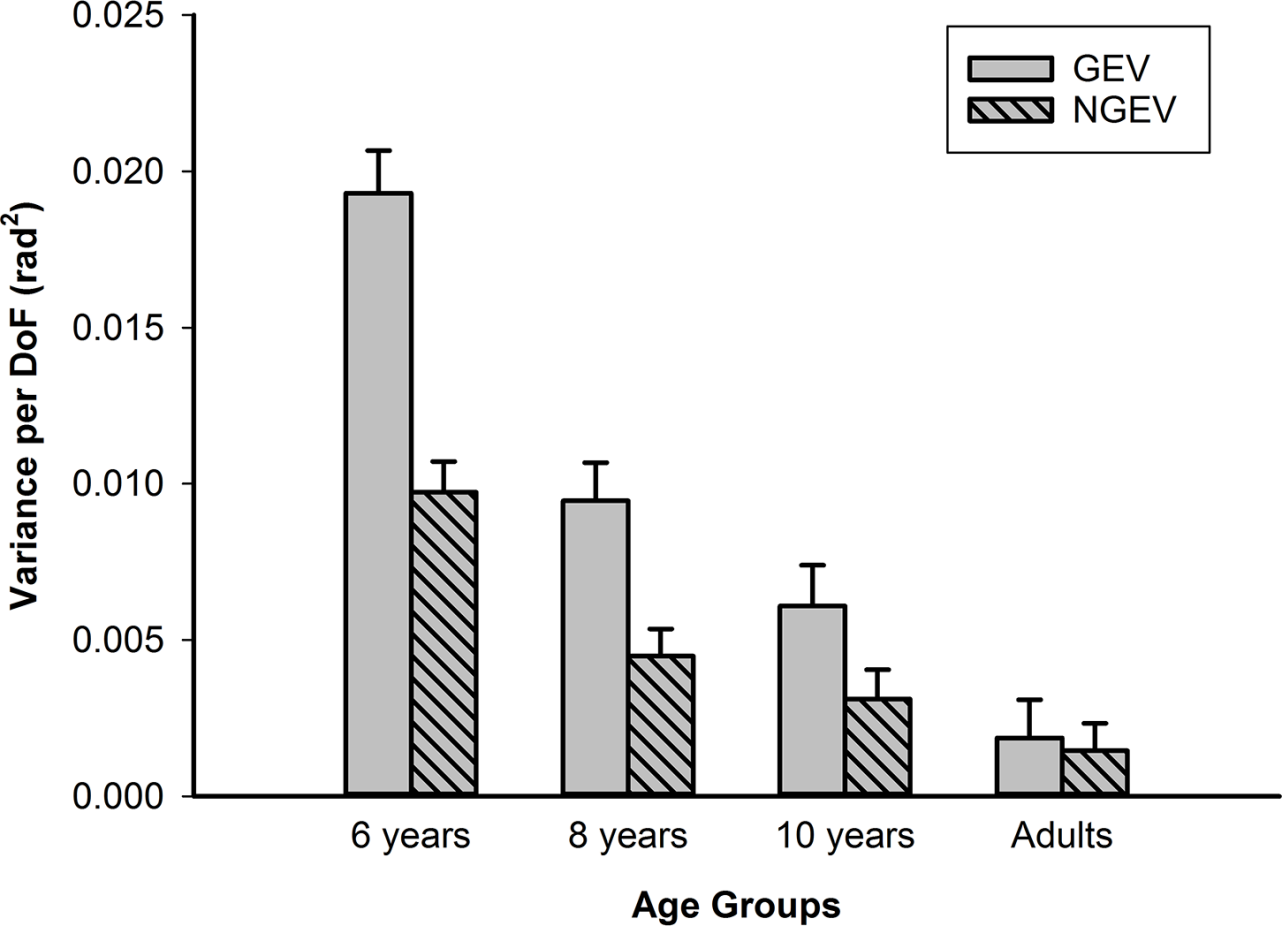
GEV (grey bars) and NGEV (dashed grey bars) at movement termination for all age groups. The mean of the vision and no-vision condition is presented. Error bars represent SEM.

Fig 4 shows GEV and NGEV values of the moment of movement termination for each age-group. The repeated measures ANOVA revealed a significant main effect for variability, *F*(1,51) = 164.47, *p* < .001, *η*^*2*^_*G*_ = 0.29, indicating lower values of NGEV than GEV. The main effect of age was also significant, *F*(3,51) = 53.13, *p* < .001, *η*^*2*^_*G*_ = 0.64, indicating changes over age. Different developmental trends for GEV and NGEV were shown by a significant interaction effect of age-group with variability, *F*(3,51) = 6.04, *p* = .001, *η*^*2*^_*G*_ = 0.04. To better describe this interaction, we computed separate linear regression lines with age-group as independent variable and GEV and NGEV as dependent variables. A significant regression equation was found for both GEV (*F*(1,53) = 82.64, *p* < .001) and NGEV (*F*(1,53) = 36.04, *p* < .001), with an *R*^*2*^ of 0.61 and 0.41, respectively. Importantly, the slope for the regression line of GEV was -0.78, whereas the slope of NGEV was -0.64. We interpreted this as showing that the decrease over age of GEV was steeper than the decrease for NGEV. No effects for vision were found.

To check that the findings at movement termination did not originate from differences at movement initiation, we compared GEV and NGEV at movement initiation and termination. The repeated measures ANOVA for GEV revealed a significant group (*F*(3,51) = 43.93, *p* < .001, *η*^*2*^_*G*_ = 0.61) and time (*F*(1,51) = 39.83, *p* < .001, *η*^*2*^_*G*_ = 0.22) effect. The repeated measures ANOVA for NGEV also revealed a significant group (*F*(3,51) = 38.19, *p* < .001, *η*^*2*^_*G*_ = 0.55) and time (*F*(1,51) = 26.50, *p* < .001, *η*^*2*^_*G*_ = 0.12) effect. Importantly, for both GEV (*F*(3,51) = 4.49, *p* = .007, *η*^*2*^_*G*_ = 0.08) and NGEV (*F*(3,51) = 5.45, *p* = .002, *η*^*2*^_*G*_ = 0.08) a significant interaction effect between time and group was found, indicating that the effects at movement termination did not simply follow from effects at movement initiation, and that this differed over age groups. Mean (*M*) and standard error (*SEM*) of variability per DoF (rad^2^) for each age group for GEV at movement initiation were: 6-year-olds *M* = 0.0059, *SEM* = 0.0009; 8-year-olds *M* = 0.0058, *SEM* = 0.0008; 10-year-olds *M* = 0.0060, *SEM* = 0.0008; Adults *M* = 0.0016, *SEM* = 0.0007. Mean (*M*) and standard error (*SEM*) for each age group for NGEV at movement initiation were: 6-year-olds *M* = 0.0033, *SEM* = 0.0003; 8-year-olds *M* = 0.0032, *SEM* = 0.0002; 10-year-olds *M* = 0.0027, *SEM* = 0.0002; Adults *M* = 0.0012, *SEM* = 0.0003.

## Discussion

The current study focused on the development of reaching during mid-childhood. Inspired by the DS approach, we studied developmental trends not just at the performance level, as commonly done in earlier studies, but also at joint angle level. Our results showed different statistical contrasts significant at different levels (performance vs joint angle), which implies different developmental trends at each level, indicating the importance of focusing on different levels for achieving understanding of reaching development. For the performance of the index finger we found a linear and quadratic contrast for VE. CE showed a linear contrast. For the joint angle level, we found linear contrasts for all joint angles, and also cubic contrasts for shoulder joint angles. No vision effect was found at the performance level nor the joint angle level. By examining the structure in joint angle variability with the UCM method, we were able to relate the performance level and variability at the joint angel level. Both variability measures, GEV and NGEV, decreased with age. Importantly, the decrease of GEV was steeper than the decrease of NGEV, revealing that the structure in joint angle variability changed as a function of age. Finding different developmental trends at different levels, as we did, not only provides new findings with regard to reaching development, but also presents us with the challenge of integrating these findings. This is done in the following where a first step is made to present a level-overarching explanation of reaching development in mid-childhood.

Before doing so, we first focus on the manipulation of vision availability, which was done to examine how this environmental constraint affected reaching and whether this differed over age. Results of the current study showed no effects of the vision manipulation at either level we studied. This is in contrast with some previous studies focusing on the performance level, which revealed more reaching errors in the no vision condition and different developmental trends across vision conditions [2,13,36,45]. The most prominent effect found was a performance decrease in 8-year-old children in the no vision condition, but not in the vision condition [2,13,46,47]. Studies finding this effect of vision on the developmental trend explained it in terms of changes in feedback and feedforward processes: 6-year-olds supposedly use feedforward processes, whereas 8-year-olds use feedback processes, while these processes would be integrated in 10-year-olds [2]. That we did not find effects of vision might be due to differences in experimental settings. For example, these earlier studies covered the movement trajectory with a horizontal screen, or used special glasses to perturb visual feedback. We, on the other hand, dimmed the light. This is where the DST can contribute in understanding these differences. By considering that task constraints might influence the result of the interaction between the involved components, the DST can explain why different experimental setups result in different developmental trends. The results suggest that vision was not a limiting constraint in the current study, that is, it did not affect the result of the interaction of the involved components. Future studies should focus on the performance decrease around 8 years in relation with vision and other task constraints.

Inspired by the DS perspective, we focused on different levels involved in reaching, because its starting point is that over development each component of the system changes on its own time-scale. Hence, if one or multiple components change, the behavior might change [16–18,48,49]. Our results revealed differences in the trends at the performance level and the joint angle level. At the performance level, we found a linear and quadratic contrast for VE, indicating a large decrease from 6- to 8-year-old children and similar values from 8-year-olds to adults, which is in line with other studies [1,11,50]. CE showed a linear contrast (marginally significant), indicating that small fine-tuning changes occur for CE. Movement time also revealed a linear contrast, suggesting a linear decrease with age. Some studies have found a linear decrease [3,51,52], but most others have found a non-monotonic trend for MT, especially in the no-vision condition [2,13,45]. Not finding a non-monotonic trend in the current study could be related to our visual conditions as discussed above. We found similar slopes of the speed-accuracy tradeoff across age-groups. This shows that speed and accuracy were not traded differently over age, showing that spatial variability measures were not confounded by differences in speed-accuracy trade-off. At the joint angle level, we found linear contrasts for the joint angle SD of all joint angles, and, importantly, also cubic contrasts for the joint angles of the shoulder. Thus, our results indicate different developmental trends at different levels, which is line with Schneiberg et al. [10], showing that different components contributing to reaching, such as the endpoint performance, joint excursions, trunk involvement, and multi-joint coordination, develop differently. The indication of finding different developmental trends at different levels underlines the relevance of understanding the developmental processes that occur at the joint angle level.

Increasing understanding about of multi-joint coordination in mid-childhood can be achieved by elaborating on what the changes in the structure of variability over age could mean. First of all, both, GEV and NGEV decreased with age. We suggest that the decrease could be related to changes in the general stability of children´s system. During mid-childhood, all components of the body are continuously changing. For example, body proportions such as mass and length fluctuate [53], postural control accompanying reaching movements develops [54], attention and executive functions change [55,56] and neurological changes occur [57]. These individual components all develop non-linearly and their interaction changes also continuously, which might result in a less stable system. An unstable system is probably reflected in higher NGEV. But an increased GEV could also indicate an unstable system, as exploiting a large range of appropriate joint angle solutions that do not affect the endpoint, might be necessary for successful reaching. This is in line with a study on adult reaching in which young adults increased GEV when performing reaching movements under uncertain task conditions, i.e., external instability [58]. Thus, if we assume that the stability of the system increases during mid-childhood development, NGEV and GEV probably decrease which could explain our findings. Future studies could test this hypothesis by letting children reach under uncertain task conditions, like it has been done in the study of de Freitas et al. [58]. If the difference between GEV values in a certain and an uncertain condition increases in magnitude with increasing age, it would suggest that compared to older children and adults, younger children do not need to further increase the range of solutions because their baseline range of solutions used is already high.

Interestingly, our results indicated that the decrease of GEV and NGEV differed. To understand the developmental trends of GEV and NGEV it might be useful to introduce the concept of synergies. A synergy can be defined as the organization of joint angles into a task-specific unit [59], representing a family of solutions for a task [60,61]. The concept of synergies is useful because it joins the performance level and the joint angle level, that is, synergies are organized according to the task at the performance level while emphasizing how the performance is brought about by the joint angles. Different than other studies, we interpret GEV as the variability that belongs to the synergy (i.e., solutions for the task), and NGEV as the variability that lays outside the synergy as it does not represent a solution of the task. In line with this interpretation, our findings with regard to GEV and NGEV showed that variability inside the synergy decreased more than variability outside the synergy which we propose could be related to exploratory behavior.

Exploration could be necessary to discover the family of solutions belonging to the synergy. This is needed to develop the ability to remain flexible and skilled in the face of inevitable and often unpredictable perturbing forces arising internally from the body or externally from the environment. This idea is related to an interpretation of Thelen et al. [22] who studied infants longitudinally over the period when they transition from not reaching to reaching. Results revealed a period of apparent loss of trajectory control after infants already had achieved controlled reaching. Thelen et al. [22] interpreted this period as a period of heightened exploration of reaching speeds, allowing infants to discover a more globally stable metric for reaching speeds. Our results could be interpreted in a similar light. Here, exploration is the search for the families of solutions belonging to a synergy. We suggest that during mid-childhood the synergy is refined based on those exploration processes, which on the one hand is reflected in a gradual decrease of error around the target and on the other hand is reflected in the specific decreases of GEV and NGEV. Most of the exploration in younger children occurs inside the synergy and this exploration rapidly decreases over age. However, searching for the family of solutions belonging to the synergy requires exploring at the boundary of the synergy. It could be possible that older children search closer to the boundaries of the synergy, implying more occasions of searching outside the synergy, which could explain why NGEV changes at a slower pace over development than GEV.

The current study has some limitations. With the current data, it is not possible to establish the trial to trial search that characterizes exploration, because the UCM is estimated for a block of trials. Future studies could conduct a learning experiment with multiple blocks [62] to assess how GEV and NGEV change over these blocks. To better understand the search from trial to trial, different analysis techniques could be used such as described in [63–65]. Also, reaching is a rather simple task. It is questionable how much exploration can actually occur in this task. However, taking the developmental changes at the performance level into account, it seems that the task is still difficult enough, otherwise no developmental changes should have been seen. Another limitation of the current study is that the UCM method was only used to analyze variability at the beginning and end of the movement and not throughout the movement.

To conclude, our results showed that developmental trends at the performance level (statistical linear and quadratic trends) and the joint angle level (statistical linear and cubic trends) differed, indicating that it is important to describe the developmental trends of different levels relevant in reaching, like it is suggested by the DS perspective. Relating these two levels brought us to the concept of synergies. We suggest that over mid-childhood development, synergies are refined through exploration. These exploration processes are reflected in a gradual decrease of errors around the target, but more importantly, they are also reflected in changes of variability outside and inside the synergy. By that we indicated that is it important to understand the processes occurring on other levels than the performance level to give a level-overarching explanation of reaching in mid-childhood.

## Supporting information

S1 File Kinematics, joint angle standard deviation and UCM measures. The second tab gives the explanation of the abbreviations.
(XLSX)
